# Auditory–tactile presentation accelerates target detection in a multitasking situation

**DOI:** 10.1186/s41235-025-00664-z

**Published:** 2025-08-22

**Authors:** Angelo G. Gaillet, Clara Suied, Gabriel Arnold, Marine Taffou

**Affiliations:** 1 Institut de Recherche Biomédicale des Armées, Brétigny-sur-Orge, France; 2 CAYLAR SAS, Villebon-Sur-Yvette, France; 3 Fédération ENAC ISAE-SUPAERO ONERA, Université de Toulouse, Toulouse, France

**Keywords:** Multisensory enhancement, Tactile, Auditory, Audio-tactile, Detection task, Multitask, Divided attention, MATB-II, 3D sound

## Abstract

There is ample evidence from cognitive sciences and neurosciences studies that multisensory stimuli are detected better and faster than their unisensory counterparts. Yet, most of this work has been conducted in settings and with protocols within which participants had the sole detection task to perform. In realistic and complex environments, such as military ones, detection of critical information has to be performed while the operator is concurrently managing several others tasks and processing a vast amount of sensory inputs. To date, it remains to determine whether multisensory benefits for detection hold true in complex multitasking situations. In the present study, we compared the detection performance of healthy participants when the target was only auditory, only tactile, or both auditory and tactile. Detection performance was measured in a simple detection task condition and in a multitasking condition. In the latter, participants had to detect the targets while concurrently performing the subtasks of the MATB-II environment, designed in the 90s by NASA to simulate piloting tasks. Multisensory acceleration of reaction times was larger during multitasking compared to single-task conditions. Crucially, participants detected auditory–tactile targets faster than their unisensory counterparts. While previous studies have reported such facilitation effects in single-task contexts, our results show that multisensory facilitation of detection speed does occur in a realistic multitasking environment and is larger than in simple task conditions. Auditory-tactile displays seem to have the potential to enhance information presentation and could be used in applied settings like military aviation.

## Introduction

Since the 1990s and the pioneering work of Meredith and Stein (Stein & Meredith, 1993), numerous studies on humans have evidenced that simultaneous presentation of information through multiple sensory modalities, such as vision, hearing and touch, can result in significant perceptual and behavioral gains (see Alais et al., 2010; Calvert et al., 2004 for reviews). Findings usually revealed faster behavioral responses to multisensory redundant signals as compared to their unisensory counterparts, provided that they are close both temporally and spatially (*e.g.*, Diederich & Colonius, 1987; Gondan et al., 2004; Miller, 1982; Molholm et al., 2004; Ngo & Spence, 2010; Suied et al., 2009). In particular, auditory–tactile redundant stimuli have been observed to accelerate responses in simple detection tasks (Gondan et al., 2004; Hecht et al., 2008a, 2008b; Murray et al., 2005; Tajadura-Jiménez et al., 2009a; Zampini et al., 2007). All these studies showed a redundant signal effect: the detection of auditory–tactile stimuli being faster than the one observed with either auditory or tactile signals alone. Furthermore, they observed that the multisensory acceleration of detection can exceed the effect that could be attributed to simple statistical facilitation linked to the availability of two sensory cues (the so-called Race Model, Miller, 1982; Raab, 1962), suggesting the integration of the cues.

The interest in auditory and tactile information displays has grown within the field of human-system interface design (De Barros & Lindeman, 2013; Ho et al., 2006; Sarter, 2006; Spence & Ho, 2008; Tannen et al., 2004). In military aeronautical environments, the safety of the crew and the success of an operation require the operators to rapidly and efficiently detect crucial information, such as alarm signals, from the interfaces. With the visual modality being already overexploited (Bourgeon et al., 2023), the use of auditory–tactile multisensory signals represents a promising solution to enhance the design of cockpits interfaces. Ho et al. (2007) examined the multisensory facilitation of warning signals detection (perceiving the brake lights of the preceding vehicle) in a simple driving scenario and reported faster responses with auditory–tactile warning signals. In this study, like in the other studies on auditory–tactile detection facilitation conducted in more controlled settings, the detection task was the participants’ sole task. In contrast, during flight, pilots face complex multitasking situations. These situations could be operationalized as divided attention tasks involving multisensory stimuli (with either visual, visuo-motor or auditory tasks) and inducing high attentional load.

In the existing literature, studies exploring the links between multisensory processes and attentional load remain sparse. There is evidence suggesting that orienting of attention toward a target stimulus presented among a vast amount of distractors can be facilitated by multisensory integration processes (Van der Burg et al., 2008). Furthermore, several studies using a rapid serial visual presentation task (RSVP; see the seminal paper by Potter & Levy, 1969) to induce high perceptual load suggested that multisensory audiovisual and auditory–tactile task-irrelevant stimuli are more efficient in capturing spatial attention in high-load conditions (Ho et al., 2009; Lunn et al., 2019; Santangelo & Spence, 2007; Santangelo et al., 2008). According to the perceptual load theory, attention works with a limited perceptual capacity. When perception is deeply taxed by a main primary task, the processing of a distractor, or of a stimulus less relevant for the main task, may decrease (Lavie, 2010). Thus, under selective attention and high perceptual load conditions, the advantage of multisensory task-irrelevant stimuli for capturing attention might lead to their processing being facilitated compared to unisensory ones.

It is also important to consider that top-down directed attention seems, in turn, to modulate multisensory integration phenomena (Alsius et al., 2005; Michail & Keil, 2018; Saccani et al., 2025; Talsma et al., 2010). It therefore seems essential to evaluate whether attending to multisensory stimuli modifies the multisensory benefits in high attentional load conditions. Lunn and colleagues examined the attentional capture of multisensory auditory–visual stimuli when the latter were task-relevant and thus were allocated some top-down attention within a dual-task paradigm. Participants performed a RSVP task. Concurrently, they had to detect and discriminate the lateral position (left or right) of a target that was either visual, auditory or audiovisual. Results revealed faster responses to audiovisual stimuli, suggesting that multisensory stimuli also enhance attentional capture under divided attention conditions (Lunn et al., 2019). Whether this is also the case with auditory–tactile stimuli remained to be tested. Moreover, in the study of Lunn and colleagues, the attentional load was manipulated by using a single unisensory visual task. During flight, pilots face much more complex situations requiring detection of critical information while concurrently piloting, communicating, monitoring the aircraft’s systems and executing the mission. These tasks are multiple and deliver information through both the visual and auditory sensory channels. To our knowledge, there is to date no study that investigated multisensory facilitation of detection under divided attention conditions which implicate more than one sensory modality.

In the present study, we investigated whether auditory–tactile redundant presentation improves target detection in a multitasking situation with attention divided over multiple tasks and sensory modalities. We used 3D sound rendering technology and a vibrotactile belt, both of which have been considered for deployment in military aviation (Miller et al., 2019; Morcos et al., 2023; Sarafian et al., 2009; Szoboszlay et al., 2021), to deliver auditory and tactile stimuli to healthy participants in a way that more closely resembles real-world situations. We measured participants’ reaction time and omission rate to target stimuli presented via the auditory, the tactile or both the auditory and tactile sensory channels. We analyzed auditory–tactile stimulation effect on detection speed by examining redundant signal effects on mean reaction times as well as the statistical facilitation on reaction times’ distributions, that also allow scrutinizing how multisensory stimuli impact RTs from early to late ones (Giray & Ulrich, 1993; Gondan et al., 2004; Hecht et al., 2008b; Juan et al., 2017; Lunn et al., 2019; Miller, 1982; Tajadura-Jiménez et al., 2009a; Zampini et al., 2007). Participants performed the detection task in two conditions: a single-task condition where the participants’ sole task was to detect the targets and a multitasking condition in which they had to perform the detection task concurrently with the different subtasks of the Multi-Attribute Task Battery (MATB-II), which involve visual and auditory sensory information. MATB-II is a tool that simulates four distinct subtasks (tracking, system monitoring, communications and resource management) reflecting activities involved in aircraft piloting (see Comstock & Arnegard, 1992 and Santiago-Espada et al., 2011 for the original descriptions of this tool and Cegarra et al., 2020 for the version used in the present study). It has been suggested that auditory–tactile facilitation effects may be influenced and constrained by spatial factors (Murray et al., 2005; Tajadura-Jiménez et al., 2009a; Zampini et al., 2007). To further pursue this line of investigation; we also examined the influence of auditory and tactile stimuli spatial congruence on detection performance by presenting them either on the same side or on opposite sides of participants’ body.

## Methods

### Participants

Twenty-five healthy participants (13 females; age: *M* = 33.6 years, *SD* = 8.8, *range* = 20–50) were recruited. Sample size was decided a priori based on previous work using similar detection tasks (Hecht et al., 2008b; Lunn et al., 2019; Zampini et al., 2007). Twenty-four were right-handed, one was ambidextrous. All had normal hearing with pure tone audiometry thresholds at 500 Hz, 1 kHz, 2 kHz, 4 kHz and 8 kHz being less or equal to 25 dB HL in each ear. They all reported normal touch. None of them reported a history of psychiatric disorders, neurological disorders or was currently undergoing medical treatment. They all gave written informed consent to participate in the study, which was approved by the Ethics Committee of the French Armed Forces Health Service (C2E DFRI-SSA—IRB00013918). Participants were compensated €30 for their participation.

### Apparatus

Participants sat on a stool at a desk in front of a 22″ computer screen, a keyboard and a Thrustmaster T. Flight Stick X joystick, in a 4*m*2 soundproof booth. They were equipped with Beyer Dynamics DT 770 PRO 250 Ohm headphones and with a tactile belt purpose-made by CAYLAR SAS. The vibrotactile belt could deliver vibratory stimuli at different positions around the waist via a set of four cylindric eccentric rotating mass vibrators positioned orthogonally to the skin of the participant. The vibrators were 15 mm long and 6 mm in diameter. At each position, the four vibrators were arranged as a 2 × 2 cm square with 14-mm center-to-center spaces. The textile envelope of the belt was made of a fabric composed of elastane and polyamide. The belt was positioned around the waist of the participants, at the height of their umbilicus. There was only one layer of light clothing between the participant’s skin and the belt. Sound stimuli were presented through a Tucker Davis Technologies RX8 Digital Signal Processor (DSP), at a 48.8 kHz sampling rate. The joystick, used to respond to the target stimuli of the detection task, was rewired to the DSP to avoid timing errors due to USB polling when collecting participants’ reaction times (RTs). This setup allowed us to measure RTs with an accuracy of less than 1 ms. A computer program written in Python 3.9.10 and running on the Windows 7 operating system was used to execute the experimental blocks and present the MATB-II tasks to the user.

### Detection task stimuli

Auditory stimuli consisted of a burst of white noise, played at a volume of 64 dBA. They were processed through binaural rendering using nonindividual Head-Related Transfer Functions (HRTFs MIT KEMAR — normal pinna; Gardner & Martin, 1995). This procedure allows to manipulate the sound source virtual location by rendering accurate auditory cues such as frequency spectrum, intensity and inter-aural differences. The spatial location of auditory stimuli was manipulated to create auditory stimuli located in the rear space (at one-meter distance from the participant’s head center) either on the left (-135°), or on the right (+ 135°) hemispace at ear level. The tactile stimulus consisted of a pulse-like vibration periodic at 125 Hz. It was presented via the vibrotactile belt on the back of the participant, at the same azimuth as the auditory stimuli, either on the left (-135°) or on the right (+ 135°) hemispace. Tactile stimuli intensity was the same for all participants and was chosen prior to the experiment to produce a vibration that was felt but not heard by participants. Both auditory and tactile target stimuli had a duration of 200 ms with a sinusoidal ramp-up and ramp-down of 30 ms and 40 ms, respectively.

### MATB-II tasks and configuration

**Fig. 1:**
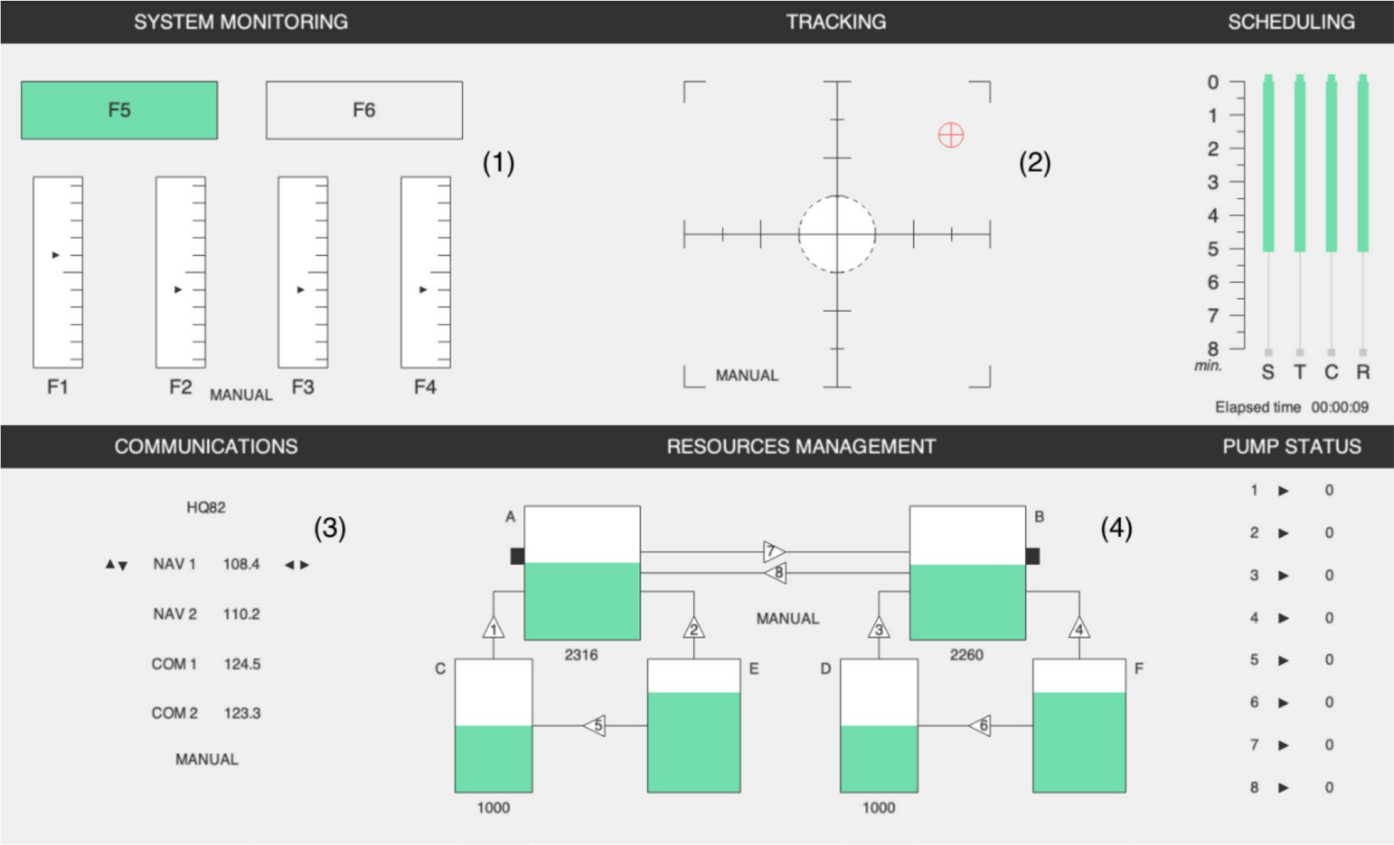
Screenshot of Open-MATB, the open-source version of MATB-II (Cegarra et al., 2020). The screenshot shows the latest version of the software which is released under CeCILL v2.1 license. Four tasks are concurrently presented to participants: system monitoring (1), tracking (2), communication (3) and resource management (4)

The MATB-II environment (Comstock & Arnegard, 1992; Santiago-Espada et al., 2011) was designed to provide an analogue to piloting activities (tracking, system monitoring, communications and resource management), and has since its creation been widely used in human factors research (Pontiggia et al., 2024; Prasetyo, 2024). We used the open-source version of the MATB-II environment (Cegarra et al., 2020; see Fig. 1). It was presented on the computer screen positioned in front of the participants. A short description of these tasks follows (for a detailed description see Cegarra et al., 2020). Four similar scenarios were designed for the experiment (one different for each detection task block, see below), all involving the four standard tasks. Each scenario lasted 5 min and 15 s to encompass the duration of an experimental block of the detection task. The four tasks were configured as follows.

The tracking task consists in keeping the cursor in the center of the visor (see Fig. 1, panel (2)). Participants were instructed to use the joystick with their right hand to compensate the movement of the indicator on the x and y axes. This task is continuous, requiring constant input by the participant to maintain the cursor in position. The joystick force parameter was set to 2 and the cursor color to “#009900.”

The system monitoring task presents four scales and two lights (Fig. 1, panel (1)). Participants were instructed to press appropriate keys with their left hand on the keyboard when the scale indicators deviated (F1, F2, F3, or F4) and when the left light turned off (F5) or the right light turned red (F6). Within a scenario, 17 failures were presented with an inter-stimulus time varying from 15 to 25 s.

The communication task consists in managing radio channels and frequencies according to the received radio instructions (see Fig. 1, panel (3)). The participant is assigned a callsign and must only respond to messages addressed to them, ignoring all others. To accomplish this, they need to use the “up/down” arrow keys on the keyboard to choose the correct radio channel, and the “left/right” keys to adjust the frequency. The language of radio communications was set to English. Within a scenario, 14 communication events were presented (eight of which directed to the user) with an inter-stimulus time varying from 15 to 30 s. The participant callsign was randomly selected at runtime and followed the “[A-Z][A-Z]\d\d” pattern. The change of frequency to be made varied from 2 to 3 MHz.

In the resource management task (Fig. 1, panel (4)), participants must keep the level of liquid in two reservoirs at a target level. To do so they had to press keys corresponding to a set of pumps (with the corresponding numeric pad key, between 1 and 8 for the different pumps). The pumps can break randomly in time, forcing the participant to dynamically change their management strategy. In our scenarios, the target tank volume value was set to 1000 and 15 pump failures were presented. Each failure lasted between 5 and 20 s. Only one failure was presented at a time and the time between failures varied from 0 to 35 s.

### Procedure

**Fig. 2:**
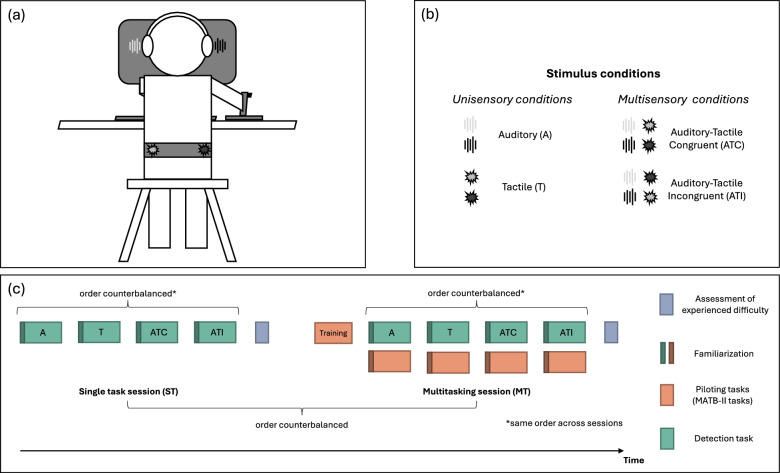
Experimental design. **a** Experimental setup. Participants sat on a stool, in front of a monitor, in a soundproof booth. They were equipped with headphones and a vibrotactile belt. **b** Stimulus conditions. Auditory (A) and tactile (T) stimuli were delivered either on the left or right side in participant’s rear space. They were either presented alone (A and T unisensory conditions) or together simultaneously (ATC and ATI multisensory conditions). In the latter case, A and T signals could either be presented on the same side or on opposite sides. **c** Example of an experimental run. The experiment consisted of two experimental sessions. In the single-task session (ST), participants had to perform the detection task only, while in the multitasking one (MT), they had to perform the detection task while also completing the four MATB-II piloting tasks. The order of the two sessions and of the sensory blocks within the sessions were counterbalanced across participants

Each participant completed two experimental sessions. In one session, participants had for sole task to perform a target detection task (single-task session, ST); in the other session, they had to perform the detection task while also completing the four MATB-II piloting tasks (multitasking session, MT). The order of the two sessions was counterbalanced across participants. Prior to the first experimental session, participants were equipped with the vibrotactile belt and the headset (see Fig. 2, panel (a)) and were familiarized with both the tactile and the auditory target stimuli of the detection task. At the end of this phase, we verified that participants did perceive the auditory and tactile stimuli position as lateralized. All participants perceived the auditory stimuli source side as well as the tactile stimuli source side correctly. None of them reported hearing the tactile stimulation.

During both experimental sessions, participants completed the detection task. They were instructed to press as fast as possible the back trigger button of the joystick when they perceived a target stimulus. Target stimuli could be auditory stimuli alone (A), tactile stimuli alone (T), spatially congruent multisensory auditory–tactile stimuli (ATC, where both auditory and tactile stimuli were presented on the same side) or spatially incongruent multisensory auditory–tactile stimuli (ATI, where auditory stimuli and tactile stimuli were presented on opposite sides—see Fig. 2, panel (a) and (b)). We measured participants’ reaction times and percentage of detected target stimuli. To avoid modality switch effects on reaction times, that is reaction times to a target stimulus being affected by the fact that the preceding target stimulus had the same or different *Sensory* modality (Gondan et al., 2004; Otto & Mamassian, 2017; Spence et al., 2001), target stimuli were presented according to their *Sensory* modality in four different blocks (A block, T block, ATC block and ATI block). Before each block, participants performed a practice block composed of four trials, to get familiarized to the sensory target stimulus. Each block was 60 target stimuli (30 left targets, 30 right targets; for the ATI block left or right representing the side of the auditory stimulus with the tactile one being presented on the opposite side) presented in a randomized manner every 2 to 8 s. The inter-stimulus intervals (ISI) were also constrained such that the total block runtime was 5 min. The presentation order of the four blocks was counterbalanced across participants, using a noncyclic Latin square, and was identical in the ST and MT sessions for a given participant (see panel (c), Fig. 2 for the description of an experimental run).

In the MT session, participants were instructed to perform all tasks (detection task and MATB-II four tasks) as well as possible and to prioritize the detection task and the MATB-II tracking task in case of difficulties. In this session, detection task stimuli and MATB-II events timing was selected independently. Participants completed the detection and piloting task with their right hand using the joystick. The other three MATB-II tasks were performed via the keyboard, with their left hand. To get familiarized with the piloting subtasks, participants underwent a 15-min training on how to carry out MATB-II piloting tasks prior to the MT session. Participants’ performance at the four piloting tasks was measured throughout the experiment. After each ST and MT session, participants were asked to give a subjective evaluation of the difficulty they faced to perform the set of tasks that had been presented to them. This was done via a vertical Visual Analogue Scale ranging from “no difficulty” to “extreme difficulty.” The selected value was stored for analysis as a floating point number ranging from 0 to 100. The experiment lasted approximately 2 h in total.

### Statistical analyses

All the statistical tests that were conducted on participants’ data were two-tailed with an alpha level of 0.05. For RT analyses, RTs shorter than 100 ms were considered as outliers and removed prior to analyses, because they were too short to correspond to a motor response to the target stimulus. In addition, RTs exceeding 1000 ms in the single-task condition were also considered outliers and removed prior to the analyses because we considered these RTs to be too long to correspond to participants correctly performing the simple speeded detection task (Suied et al., 2008). Finally, as the shortest ISI in the experimental design was 2 s, RTs longer than 2000 ms were considered as omissions.

Participants’ omission rates were analyzed to verify that they accurately performed the detection task, which was designed with sensory signals presented above detection threshold but with the intensity of tactile cues kept low so that the participants could feel but not hear them. Participants’ mean RTs were then analyzed using a repeated-measures ANOVA to examine redundant signal effects of auditory–tactile stimulation. To investigate in more detail these effects, a distributional analysis was conducted. RT distributions contain far more information than is captured by their mean values alone (Schwarz & Miller, 2012) and allow for the examination of multisensory statistical facilitation predictions on RTs (Miller, 1982; Raab, 1962).

## Results

Three participants were excluded from the analyses because their data indicated that they failed to perform the detection task with reasonable performance. Two of them missed more than 10% of the targets in one single-task condition, and the third participant missed more than 50% of targets in two multitasking conditions. A fourth participant was excluded from the analyses because they did not perform one of the MATB-II tasks in the MT condition (null score at the communications task). Therefore, the analyses were performed on the remaining 21 participants (11 female; age: *M* = 33.8 years, *SD* = 8.8, *range* = 22—49; 20 right-handed, 1 ambidextrous). Finally, 0.12% of trials had missing or incomplete data due to technical errors. These were removed before performing the statistical analyses.

### Detection task

#### Rate of omission

**Fig. 3:**
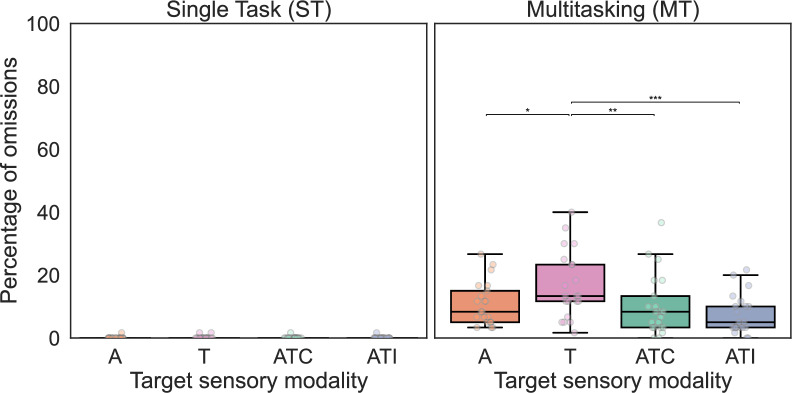
Percentage of omissions. This figure depicts participants’ (*n* = 21) percentage of omitted responses to targets in the single task (ST; left) and in the multitasking (MT; right) conditions, as a function of the *Sensory* modality of the target (auditory (A); tactile (T), auditory–tactile congruent (ATC) or auditory–tactile incongruent (ATI)). The boxes range from the first to the third quartile, within it the horizontal line denotes the median. The whiskers are based on 1.5 times the inter-quartile range. Participants missed more targets in the MT condition as compared to the ST condition. In the MT condition, there were significantly more omissions of T targets than of the others (A, ATC and ATI targets). Asterisks indicate statistically significant differences: * *p* < .05, ** *p* < .01, *** p < .001

Participants’ rate of omission was not significantly different for left as compared to right targets (paired t test, *p* = .240). Therefore, for the following analyses, data for the two *Side* conditions were pooled. On average, participants missed 0.10% of trials in single-task (ST) blocks and 11.35% of trials in multitasking (MT) blocks. As shown on Fig. 3, the rate of omissions was significantly different according to the *Task* condition (ST/MT) for each *Sensory* modality (Wilcoxon signed-rank test, *W* ≥ 190, *p* < .001, *r* = 1.00 in all cases). In ST conditions, participants’ omission rate did not significantly vary according to the *Sensory* modality of the targets (Friedman test, χ^2^_(3)_ = 0.69, *p* = .875). In the MT condition, participants’ omission rate was significantly influenced by the *Sensory* modality of the targets (Friedman test, χ^2^_(3)_ = 15.27, *p* = .002). In particular, in the MT condition, the rate of omissions for T stimuli was higher than those for ATC (Conover post hoc test with Holm correction, *T* = 3.02, *p* = .004, *r*_*rb*_ = .66), ATI stimuli (*T* = 4.25, *p* < .001, *r*_*rb*_ = *.83*) and A stimuli (*T* = 2.33, *p* = .023, *r*_*rb*_ = .61). All other comparisons were not statistically significant (*p* > .059 in all cases).

#### Reaction times (RTs)

Outlier RTs represented 0.12% of trials in the ST condition and 0.18% of trials in the MT condition. They were uniformly distributed across *Sensory* modalities (between 2 and 6 data points by *Sensory* condition) and were removed before conducting the analyses herein below.

#### Analyses of mean RTs

**Fig. 4:**
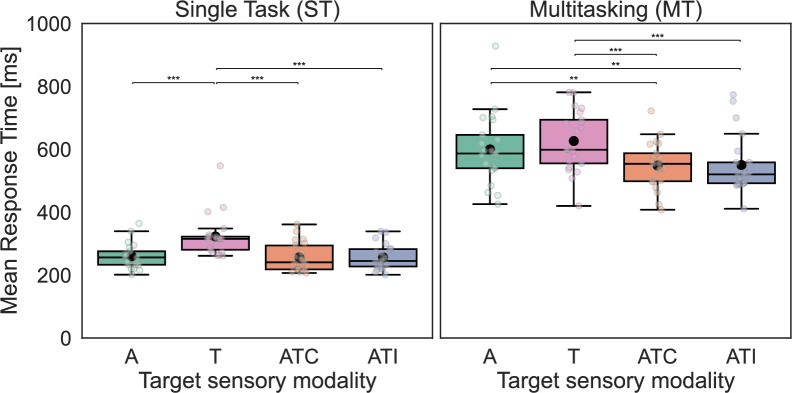
Analysis of mean reaction times. This figure depicts participants’ (n = 21) mean reaction times (RT) to targets in the single task (ST; left) and in the multitasking (MT; right) conditions, as a function of the *Sensory* modality of the target (auditory (A); tactile (T), auditory–tactile congruent (ATC), or auditory–tactile incongruent (ATI)). The boxes range from the first to the third quartile, within it the horizontal line denotes the median. The mean is represented by a black dot. The whiskers are based on 1.5 times the inter-quartile range. As expected, participants were slower at detecting targets in the MT condition than in the ST one. In the ST condition, participants detected A, ATC and ATI targets at similar speed and faster than they detected T targets. In the MT conditions, participants detected multisensory ATC and ATI targets faster than unisensory A and T targets. Asterisks indicate statistically significant differences: * *p* < .05, ** *p* < .01, *** p < .001

The distribution of measured RTs exhibited skewness, a common feature in reaction time data (for an example see Ulrich & Miller, 1993). To address this, we applied a natural logarithm transformation before proceeding with subsequent analyses (Whelan, 2008). We then calculated mean RTs to target stimuli for each participant, each *Task* and *Sensory* condition separately (for a representation of mean RTs without log-transformation see Fig. 4). The resulting values met the assumptions to perform an ANOVA (Howell, 2010). In particular, Q-Q plots indicated that residuals followed a normal distribution and Mauchly’s test (Mauchly, 1940) indicated no violation of sphericity for either the *Sensory* factor (χ^2^_(5)_ = 3.38, *p* = .642) or Task*Sensory (χ^2^_(5)_ = 7.47, *p* = .188).

A repeated-measures ANOVA was performed on the mean log-transformed RTs with the within-subject factors of *Task* (ST, MT) and *Sensory* modality (A, T, ATC, ATI). The main effect of *Task* was significant (*F*_(1,20)_ = 322.59, *p* < .001, η^2^_p_ = .94) indicating, as expected, that mean RTs were shorter in the ST condition (*M* = 274 ms, *SD* = 56 ms) than in the MT condition (*M* = 581 ms, *SD* = 100 ms). This effect was verified for each *Sensory* conditions (p < .001 and *Cohen’s d* ≥ 3.65 in all cases). We also observed a significant main effect of *Sensory* modality (*F*_(3,60)_ = 58.37, *p* < .001, η^2^_p_ = .74) and of the interaction between *Task* and *Sensory* modality (*F*_(3,60)_ = 9.52, *p* < .001, η^2^_p_ = .32). This indicates that the multitasking condition influenced participants’ RT as a function of the target *Sensory* modality. A post hoc sensitivity analysis using G*Power (Faul et al., 2007) indicated that the experiment had an 80% power to detect effects of at least *f* = 0.29 (which corresponds to η^2^_p_ = .08). All observed effects for this ANOVA exceeded this threshold, supporting the adequacy of the sample size for its findings. Given the significant results of the ANOVA, we performed post hoc t test with Holm correction (Holm, 1979).

As seen on the left part of Fig. 4, in the ST condition, mean RTs to ATC (*M* = 256 ms, *SD* = 46 ms) and ATI (*M* = 256 ms, *SD* = 41 ms) stimuli were significantly shorter than those to T (*M* = 323 ms, *SD* = 65 ms) stimuli (*p* < .001 in both cases, *Cohen’s d* = 1.47 and 1.44, respectively). However, mean RTs to ATC and ATI were not significantly different from those to A stimuli (*M* = 260 ms, *SD* = 41 ms) stimuli (*p* = .833 and *p* = .850, respectively). Furthermore, no significant difference was found between ATC and ATI (*p* = .850). Finally, mean RTs to T stimuli were significantly longer that those to A stimuli (*p* < .001, *Cohen’s d* = 1.35). In summary for the ST condition, participants detected A, ATC and ATI targets at similar speed and faster than they detected T targets.

As seen on the right part of Fig. 4, in the MT condition, mean RTs to ATC stimuli (*M* = 548 ms, *SD* = 75 ms) were significantly shorter than those to A (*M* = 599 ms, *SD* = 113 ms) and T stimuli (*M* = 626 ms, *SD* = 97 ms) (*p* = .004 and *p* < .001, *Cohen’s d* = 0.62 and 0.89, respectively). This was true also for mean RTs to ATI stimuli (*M* = 550 ms, *SD* = 94 ms) which were found to be significantly shorter than those to A and T stimuli (*p* = .005 and *p* < .001, *Cohen’s d* = 0.59 and 0.86, respectively). Finally, in MT conditions, no significant difference was found between the A and T modalities (*p* = .195) or between the ATC and ATI ones (*p* = .844). In summary, in the MT condition, participants detected multisensory ATC and ATI targets faster than unisensory A and T targets. These results indicate a redundant signal effect (RSE; Kinchla, 1974) with the multisensory targets (spatially congruent and incongruent) detected significantly faster than their unisensory counterparts.

In order to study the effect of auditory and tactile signals spatial congruence on multisensory targets detection, we presented the auditory and tactile stimuli either on the left or the right side of the participant. The side of target presentation was not a factor of primary focus in the current study. However, we performed an analysis to verify that it did not interact with the *Sensory* factor effect, which was our factor of interest. We performed a repeated-measures ANOVA on the mean log-transformed RTs, with the within-subject factors of *Side, Task* and *Sensory.* For this analysis, only the A, T and ATC modalities were considered, as discussing targets side with regard to the spatially incongruent stimuli held no meaning. A post hoc sensitivity analysis indicated that this ANOVA had an 80% power to detect effects of at least *f* = 0.27 (which corresponds to η^2^_p_ = .07). Coherently with the previous analysis, we found a strong effect of *Task* (*F*_(1,20)_ = 297.11, *p* < .001, η^2^_p_ = .94), of *Sensory* (*F*_(2,40)_ = 65.53, *p* < .001, η^2^_p_ = .77) and of the two-way interaction Task*Sensory (*F*_(2,40)_ = 12.84, *p* < .001, η^2^_p_ = .39). We also observed a significant main effect of *Side* (*F*_(1,20)_ = 7.14, *p* = .015, η^2^_p_ = .26), with mean RTs to targets presented on the right side being shorter than to those presented on the left side. The two-way interaction Side*Task was also significant (*F*_(1,20)_ = 8.75, *p* = .008, η^2^_p_ = .30), with mean RTs to stimuli presented on the right being significantly shorter than to those presented on the left side in the MT condition but not in the ST condition. Participants had to press a joystick trigger with their right hand when they detected a target stimulus. Thus, the advantage of the right side presentation of targets that we observed could be explained by the well-known “stimulus–response spatial compatibility” effect (Simon & Rudell, 1967; Umiltá & Nicoletti, 1990), in which RTs to stimuli are shorter when located in the same part of space as the required response. In the MT condition, this effect may be emphasized by attentional effects (Klein & Ivanoff, 2011; Simon & Rudell, 1967).

Importantly, there was no significant interaction between the effects of the *Side* and *Sensory* factors (*F*_(2,40)_ = 0.75, *p* = .479, η^2^_p_ = .30). The multisensory presentation effect on detection was not influenced by the side of targets presentation. For this reason, together with the fact that stimulus presentation *Side* was not one of the variables of interest in our experiment, we pooled the data from the two *Side* conditions in the RT analyses.

Finally, we explored whether the multisensory facilitation of detection speed was influenced by the relative timing between the presentation of detection task stimuli and of MATB-II communication subtask stimuli, that both deliver auditory information. To do so, we classified RTs to detection task stimuli as being presented within or outside windows of concurrent communication stimuli. RTs data, for which we could not be certain that they were deliver in the presence of a communication stimulus, were removed from the analyses (communications in MATB-II can have a variable duration and the exact duration of each was not recorded). RT data available for the analysis represented 81.54% of the RT measures remaining after removal of misses and outliers. RTs were then log-transformed and averaged by participant and condition, similarly to what was done for the other analyses. A repeated-measures ANOVA showed an effect of the *Sensory* (*F*_(3,60)_ = 13.03, *p* < .001, η^2^_p_ = .39) and *Communication presence* (*F*_(1,20)_ = 11.91, *p* = .003, η^2^_p_ = .37) factors. In fact, RTs were significantly longer in the presence of a concurrent communication (*M* = 581 ms, *SD* = 116 ms) than without (*M* = 558 ms, *SD* = 119 ms). Importantly, the analysis did not show any significant effect of the interaction of the two factors (*F*_(3,60)_ = 0.17, *p* = .915, η^2^_p_ = .009). This indicates that the presence of the communication task soliciting the auditory channel did not influence detection speed differently according to the *Sensory* modality of the target. Rather, in the presence of the communication task, target detection was globally longer. This is probably due to the additional attentional and perceptual load on the participant.

#### Analyses of the distribution of RTs

To investigate in more detail the RTs, we conducted a distributional RT analysis. Its aim was to assess whether the mean differences identified in the previous analysis between the detection speed in response to unisensory and multisensory targets were supported by differences in the distribution of RTs. We used the algorithm and MATLAB code described by Ulrich and colleagues (Ulrich et al., 2007) to compute the average cumulative distribution function (CDF) of RTs for each *Sensory* and *Task* condition tested. First, empirical CDFs were estimated for each participant. Percentile values were then computed from these empirical CDFs, using ten bins with edges ranging from the 5th to the 95th percentile, increasing in steps of 10 percentiles. The resulting RT percentile values for each of the ten bins were aggregated across participants to obtain the average CDF, reflecting the distribution of participants’ RTs. Then, to compare RT distributions between *Sensory* conditions, we performed bin by bin paired t tests on RT percentile values for ST and MT conditions separately, and applied a Benjamini–Hochberg correction to control for multiple testing (Benjamini & Hochberg, 1995).

**Fig. 5:**
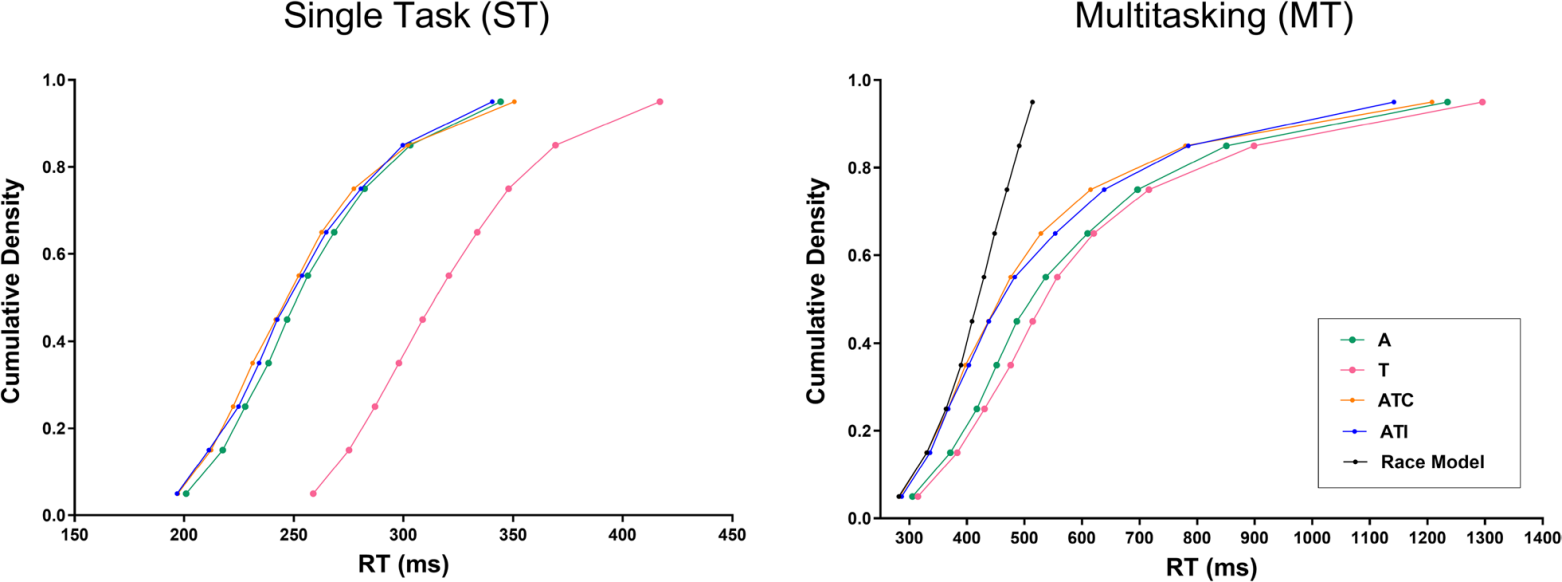
Analyses of reaction times distributions. This figure depicts the average cumulative density functions (CDFs) of participants’ (n = 21) reaction times (RTs) to targets in the single task (ST; left) and in the multitasking (MT; right) conditions, as a function of the *Sensory* modality of the target (auditory (A); tactile (T), auditory–tactile congruent (ATC) or auditory–tactile incongruent (ATI)). In the ST condition, RTs distributions were similar with A, ATC and ATI targets with globally faster RTs than with T targets. In the MT condition, RTs to multisensory targets (ATC, ATI) were markedly faster than RTs to unisensory targets with a global shift of RTs distribution. This facilitation of detection could be accounted for by a statistical facilitation as there was no violation of the Race Model

In the ST condition (see left part of Fig. 5), no significant differences were found between the CDF of RTs to A targets and the CDFs of RTs to multisensory ATC and ATI targets (*p* > .094 in all cases). In contrast, the CDF in the T condition was different from the ones in the ATC and ATI conditions, with all RT percentiles being smaller for multisensory than tactile targets (*p* < .001 in all cases).

In the MT condition (see right part of Fig. 5), CDFs of RTs to ATC and ATI targets were significantly different from CDFs of both A and T conditions. RTs distributions in the multisensory conditions were shifted leftward, with RTs percentiles being consistently smaller than the ones in each of the unisensory conditions (*p* < .001 in all cases). This result further confirms a redundant signal effect (RSE; Kinchla, 1974) in the MT condition with all RTs to multisensory targets (from early to late RTs) being faster than the ones to their unisensory counterparts.

We then tested whether the RSE found in the MT condition exceeded the effect that can be predicted by statistical facilitation using the Race Model inequality (Miller, 1982). Under the idea of a Race Model (Raab, 1962), RTs to multisensory redundant targets are controlled by the sensory signal that leads to faster detection on its own. Statistically, RTs to the race winning signal would be on average faster than the mean RT to each unisensory signal alone. Data from the literature have often shown a RSE with early RTs to multisensory redundant targets being statistically faster than the Race Model predictions. This suggests that, beyond statistical facilitation, the redundant sensory signals may co-activate and combine to lead to faster responses (Diederich & Colonius, 1987; Gondan et al., 2004; Miller, 1982; Zampini et al., 2007). To determine whether the RSE we found was larger than the one that would be predicted by the Race Model, we used the same algorithm and MATLAB code described above (Ulrich et al., 2007) to test the Race Model inequality. We computed the bounding sum of the CDFs of the unisensory A and T signals (Race Model CDF) and compared it to the CDFs in the multisensory conditions (ATC, ATI). Any RT percentiles smaller in the multisensory CDFs compared to the Race Model CDF would mean that the observed RSE can not only be accounted by a statistical facilitation but could instead be explained by sensory coactivation (Miller, 1982). As depicted in the left part of Fig. 5, we found no differences between early RT percentiles in the multisensory (ATC and ATI) CDFs and Race Model CDF. Longer RT percentiles were larger in the multisensory CDFs than in the Race Model CDF. This indicates that our auditory–tactile RSE did not invalidate the Race Model explanation, where auditory–tactile redundant signal would accelerate detection via statistical facilitation.

### MATB-II piloting tasks

#### Subjective ratings of difficulty

The difference in participants’ subjective ratings of the difficulty they experienced to perform either the detection task alone (ST condition) or both the detection and MATB-II tasks (MT condition) did not significantly deviate from a normal distribution (Shapiro–Wilk normality test, *p* = .344). As expected, participants experienced significantly higher difficulty in MT condition (*M* = 63.6, *SD* = 20.2) than in ST condition (*M* = 7.0, *SD* = 7.3, paired t test: *p* < .001, *Cohen’s d* = 2.99).

#### Performance

Participants’ performance at the piloting tasks was measured throughout the experiment. We calculated it following the method described in the documentation of Open-MATB (Valéry, 2023). In particular, performance at the discrete-event tasks (system monitoring and communications) was calculated, after each event, as the ratio of correct responses over a four-events sliding window. For the continuous tasks (tracking and resource management), performance was defined as the proportion of task frames in which the cursor was on target over the previous 5 s. Task frames (i.e., snapshots of all task parameters at a given time) were generated at the default frequency of 200 Hz for the tracking task and 0.5 Hz for the resource management task. Finally, to perform the analyses shown in the results section, the data points of each participant’s performance were averaged task by task over the duration of each block.

**Fig. 6:**
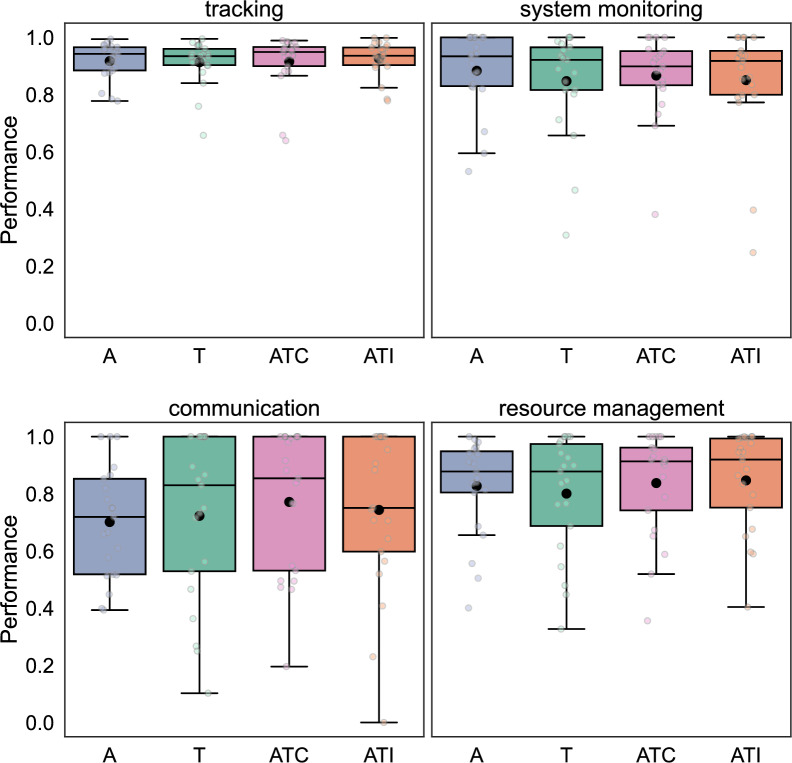
Performance at the MATB-II tasks. This figure depicts participants’ (*n* = 21) performance at each of the four MATB-II subtasks as a function of the target *Sensory* modality at the detection task. The boxes range from the first to the third quartile, within it the horizontal line denotes the median. The mean is represented by a black dot. The whiskers are based on 1.5 times the inter-quartile range. Participants’ performance suggests that they prioritized the tracking task as instructed. There was no significant effect of the *Sensory* modality of the detection task target on performance at the MATB-II tasks

No participant included in the analysis had abysmal performances at any piloting tasks for all *Sensory* modalities, suggesting that they were all committed to performing the requested tasks. As can be seen in Fig. 6, participant performance was the best for the tracking task, indicating that the completion of this task was prioritized as instructed. Participants’ performances at MATB-II tasks were not significantly modified by the *Sensory* modality of the target of the detection task (Friedman ANOVAs; *p* > .223 for each of the four MATB-II tasks).

## Discussion

We investigated whether auditory–tactile redundant presentation can facilitate target detection in humans when in a multitasking context. We used a speeded detection task and measured participants’ reaction times and omission rate to multisensory auditory–tactile targets and to the corresponding auditory and tactile unisensory counterparts. Participants performed either the detection task alone or concurrently with four different tasks simulating activities involved in aircraft piloting (MATB-II). Participants’ accuracy of detection (as assessed by omission rates) decreased in the multitasking condition and was not significantly enhanced by auditory–tactile targets. Crucially, we found that auditory–tactile signals speeded up target detection in the multitasking condition, in comparison with auditory or tactile signals alone. Moreover, participants’ performance at the four MATB-II piloting tasks were not found to be negatively affected by the multisensory targets. In other words, multisensory auditory–tactile signals facilitated detection without disrupting simulated piloting activities. In the single-task condition, however, the multisensory redundant signals did not confer such a facilitation of detection over unisensory signals.

As is commonly observed in similar studies, almost no omissions were found in the single-task condition (Gondan et al., 2005; Hecht et al., 2008a, 2008b; Murray et al., 2005; Tajadura-Jiménez et al., 2009a; Zampini et al., 2007), indicating that participants correctly performed the task and that the stimuli were sufficiently above threshold to be easily perceived. In the multitasking condition, as attention was divided between several tasks, the number of missed targets increased. This phenomenon has been evidenced multiple times in the existing literature with unisensory targets (see the seminal paper by Pashler, 1994). Some of these omissions could also be attributed to late detection and response outside the specified time window. In the multitasking condition, the omission rate to tactile stimuli was higher than that to the other modalities. This could be attributed to the tactile input intensity being relatively low as we calibrated it to be felt but not heard by the participants. When stimuli were multisensory, the number of misses dropped to the same level as for the auditory ones. These findings are coherent with the findings of Lunn and colleagues’ study, which show higher error rate in a spatial discrimination task with auditory stimuli in a divided attention setting while error rate was reduced and equivalent in response to auditory–visual and visual stimuli (Lunn et al., 2019).

This study was designed to investigate facilitation effects in terms of RTs, with its primary contribution residing within this domain. In the single-task condition, we did not observe any redundant signal effect on detection speed as participants’ responses were not faster to auditory–tactile signals than to each of the unisensory signals. The difference in the effectiveness of our unisensory signals could have played a role in this divergence. We reduced the intensity of the tactile vibratory stimulus to ensure that it remained inaudible to participants. Unsurprisingly, reaction times in the unisensory conditions show that participants were significantly slower at detecting the tactile signal compared to the auditory one, which was presented at a higher relative intensity compared to the detection threshold. The fact that, in the single-task condition, auditory signals were detected as fast as the auditory–tactile redundant signals in our study is coherent with the Race Model explanation (Raab, 1962); the reaction time to the multisensory redundant signal being controlled by the sensory signal that leads to the faster detection on its own.

The critical result that has emerged from our study is that auditory–tactile stimuli accelerated target detection in the multitasking condition, with a clear multisensory redundant signal effect. This auditory–tactile facilitation aligns with the redundant signal effect found in the literature with single detection tasks (Gondan et al., 2004; Hecht et al., 2008a; Tajadura-Jiménez et al., 2009a; Zampini et al., 2007) and goes further by suggesting that this enhancement of detection speed is not limited to controlled, over-simplified environments but extends to more ecological and complex situations. Participants’ reaction time to unisensory auditory and tactile targets were comparable when engaged in the MATB-II tasks. This is in contrast with our data from the single-task condition and could be linked to the auditory perceptual load induced by MATB-II (Wickens, 2002, 2008), which is a multisensory tool that conveys information through both visual and auditory channels. The increase in detection speediness with auditory–tactile stimuli that we found did not invalidate the Race Model explanation, where redundant sensory signal lead to faster detection via statistical facilitation. Research has shown that multisensory interactions can be modified by the spatial arrangement of the sensory signals relative to each other (Gondan et al., 2004) or relative to the perceiver’s body (Amiel et al., in revision; Canzoneri et al., 2012; Hobeika et al., 2020; Serino et al., 2015; Stone et al., 2017), as well as by stimuli semantic and affective content (Ferri et al., 2015; Taffou & Viaud-Delmon, 2014; Taffou et al., 2021). Future studies should investigate whether manipulating these stimuli features may permit to further enhance detection facilitation in multitasking situations with multisensory response times being even faster than the fastest reaction time to each of the unisensory signals. Still, in the present study, we observed an interesting behavioral effect with not only shorter reaction times on average but a global shift of reaction times distribution as compared to each of the unisensory stimuli. Previous studies showed that task-irrelevant auditory–tactile cues are particularly effective in capturing participants’ attention away from concurrent tasks (Ho et al., 2009; Santangelo et al., 2008). The facilitation of detection that we found here further highlights the importance of redundant auditory and tactile presentation in demanding contexts. Furthermore, while auditory–tactile presentation of the stimuli improved detection speed, there were no indications that it hindered the performance at the piloting subtasks (see Fig. 6) and the rate of missed targets did not deteriorate either. These findings are particularly important for applied contexts such as military aviation, in which alarms need to be perceived quickly but not at the expense of others main tasks, such as piloting the aircraft.

The increased multisensory benefit that we observed in the multitask condition may be linked to the concurrent tasks stimulating the same *Sensory* modality as multisensory targets. MATB-II tasks include the communication task, which involves auditory information. This concurrent auditory stimulation could have decreased the effectiveness of target auditory cues and lead, coherently with the inverse effectiveness principle (Diederich & Colonius, 2004; Stanford et al., 2005), to the enhancement of the redundant signal effect magnitude. However, if this were the case, we would expect to find the timing of targets presentation (i.e., whether it occurred concurrently with the audio playback of the communication subtask or not) to influence the multisensory acceleration of detection; this was not supported by our data.

The links between multisensory integration and attention have mainly been studied in selective attention settings, in which the load task is unisensory (either visual or auditory; Ho et al., 2009; Lunn et al., 2019; Santangelo & Spence, 2007; Santangelo et al., 2008). The attentional load theory posits that, when perceptual resources are heavily solicited by a main ongoing task, the processing of distractors may be diminished because fewer resources are available to process task-irrelevant stimuli (Lavie, 2010). Santangelo and colleagues propose that, in this context of selective attention, the heavy solicitation of perceptual resources by demanding tasks may also raise the threshold that task-irrelevant stimuli have to reach to capture participants’ attention and that multisensory stimuli seem more efficient in capturing attention away from the main task (Santangelo et al., 2008). In the context of divided attention, Lunn et al. (2019) reported that audiovisual task-relevant stimuli also accelerate detection, when the detection task has to be performed concurrently with a RSVP task. Our findings are coherent with this study and the hypothesis of a multisensory enhancement of attentional capture in divided attention settings. Further, we demonstrated an advantage of auditory–tactile stimuli in a complex situation involving multiple tasks (in addition to detection) and that engage different sensory modalities, among which a continuous task. This contributes to the accumulation of data, indicating that multisensory auditory and tactile stimuli are promising information display solutions in demanding contexts (De Barros & Lindeman, 2013; Ho et al., 2006; Sarter, 2006; Spence & Ho, 2008; Tannen et al., 2004). With this study settings, we measured multisensory facilitation in an ecological yet controlled protocol. It would now be interesting to explore whether the multisensory benefit increases with the number of tasks and/or according to their *Sensory* modality, or whether it reaches a maximum or even decreases at some point. This could be done by examining the auditory–tactile facilitation when participants are concurrently performing only one of the MATB-II subtasks alone and then with an increasing number of subtasks.

Noteworthily, in the multitasking situation, we observed the redundant signal effect regardless of the spatial congruence of the auditory and tactile signals. Even when presented on opposite sides of participants’ body, the presence of auditory and tactile stimulation accelerated target detection. Studies from the literature examining spatial constraints of auditory–tactile redundant signal effect in single detection tasks report divergent results depending on the body part that receives the tactile stimulation. While detection task studies delivering tactile stimulation on the participants’ hand showed similar RSE with auditory and tactile stimuli presented in same or opposite sides (left/right) (Tajadura-Jiménez et al., 2009a) or hemifields (front/back) (Zampini et al., 2007) of participants’ body, the ones delivering tactile stimulation on the head reported a weaker RSE when auditory and tactile stimuli were not spatially aligned (Murray et al., 2005; Tajadura-Jiménez et al., 2009a). This has been proposed to arise from cerebral representation of body surface being higher for the head than for other body parts (Fu et al., 2003; Tajadura-Jiménez et al., 2009a; Zampini et al., 2007). Our present results seem consistent and suggest that, at least in multitasking situations in which spatial information is task-irrelevant, auditory–tactile RSE involving tactile stimulation on participant’s lumbar region is not subject to a spatial modulation linked to stimuli left/right alignment. This could be of interest for the deployment of auditory–tactile displays that could actually be made easier when less constrained by sensory signals co-localization. Further research should also look into the spatial constraints for detection facilitation with body parts such as the hand, the torso, or the thigh, that can also be considered for tactile stimulation in aeronautical interfaces.

The fact that our simple detection task did not rely on spatial information can contribute to the redundant signal effect occurring regardless of sensory signals spatial congruence. The spatial location of our stimuli was not relevant to perform the speeded detection task. This has been already discussed for similar results with both auditory–tactile (Murray et al., 2005; Zampini et al., 2007), auditory–visual (Hughes et al., 1994; Teder-Sälejärvi et al., 2005) and visuo-tactile redundant stimuli (Girard et al., 2013). This could explain our results contrasting with those of a prior study by Ho et al. (2009) which also presented stimuli in the lumbar region but for which spatial correspondence was relevant to the primary task. Future research should explore whether task-specific spatial requirements could lead to different spatial constraints for auditory–tactile behavioral facilitation.

The simple detection task that we used in the present study was a necessary first step to examine the behavioral consequences of auditory–tactile stimulation in multitasking situations. Auditory–tactile redundant stimulation has also been reported to enhance target recognition, target search, target spatial discrimination, or target localization, when the task is performed in isolation (Godfroy-Cooper et al., 2015; Hecht et al., 2008a; Molholm et al., 2004; Ngo & Spence, 2010; Suied et al., 2009). A following step in investigating the benefits of auditory–tactile signals in multitasking conditions is to examine these processes that are also mobilized during flight operations.

## Conclusion

Auditory–tactile signals accelerated target detection in a simulated piloting task. At the same time, we did not find any evidence that performance on piloting tasks was negatively affected in this multisensory condition compared to the unisensory ones. Yet, participants were clearly engaged in the simulated flight operation tasks, as evidenced by their performances at each one, their reduced target detection performance relative to the single-task condition, and their subjective experienced difficulty. The multisensory auditory–tactile redundant signal effect seems thus particularly interesting from an applied point of view. Although more proofs are needed for this emerging topic in the literature, in light of recent work, we think that auditory–tactile presentation of information may actually even help reduce some of the cognitive load in complex tasks and environments (Marucci et al., 2021; Remigereau et al., 2024).

While it remains to be determined whether our findings will transfer to real aeronautic military environments, our results add to a growing body of research suggesting that auditory–tactile stimuli can be beneficial for information presentation in human-system interfaces (Gillmeister & Eimer, 2007; Ho & Spence, 2017; Ho et al., 2007, 2009; Santangelo & Spence, 2007). In particular, they might help by facilitating the detection of critical alerts or by helping the pilot to disengage from the piloting tasks and to engage in treating the alert. With the development and accessibility of 3D sound and tactile rendering technologies, the potential to manipulate and control the features of auditory–tactile signals display is vast. Advancing toward a more complete comprehension of auditory–tactile behavioral facilitation and its spatial, temporal and semantic determinants would provide useful insights for exploring auditory–tactile benefits in more applied settings.

## Data Availability

The conditions of our ethics approval do not permit public archiving of anonymized study data. Readers seeking access to the data should contact the corresponding author. Access will be granted to named individuals in accordance with ethical procedures governing the reuse of sensitive data and contingent upon the establishment of a formal data sharing agreement.
